# Neurological Examination: An Evaluation of Video-Based Learning

**DOI:** 10.7759/cureus.51866

**Published:** 2024-01-08

**Authors:** Giorgio Guido, Simone Franceschini, Vittorio Oteri, Matilde Pavan, Peter G Bernad

**Affiliations:** 1 Department of Biomedical and Biotechnological Sciences, University of Catania, Catania, ITA; 2 School of Medicine, University of Chieti, Chieti, ITA; 3 Endocrine Unit, Department of Clinical and Experimental Medicine, Garibaldi-Nesima Hospital, University of Catania, Catania, ITA; 4 School of Medicine, University of Milan, Milan, ITA; 5 Department of Neurology, George Washington University Hospital, Washington DC, USA

**Keywords:** youtube, video-based learning, physical examination, neurological examination, medical education

## Abstract

Objective

This study aimed to systematically review and assess educational YouTube videos on neurological examination.

Methods

YouTube was screened for educational videos on neurological examination. A scoring system (involving five major and six minor criteria) was used to assess videos. Educationally useful videos were defined as those satisfying all major criteria and at least three minor criteria; 2 points were allocated for each major criterion and 1 point for each minor criterion, thereby using a score of 13 as a threshold.

Results

A total of 500 videos were screened, and 128 videos were included in the final selection procedure. Only 55 videos were deemed as educationally useful; 13 of these videos focused on the general neurological examination, 10 on cranial nerves, 11 on the upper limb, five on the lower limb, three on reflexes, one on upper and lower limbs, one on gait, and 11 were in the form of lectures. Six (46.15%) of the educationally useful videos about general neurological exams, including the top three videos, were created by academic institutions, and three (23.07%) were book-related. Educationally useful videos were not the most viewed videos. None of the analyzed videos included the evaluation of the autonomic nervous system in the physical examination routine.

Conclusions

YouTube is an increasingly common source of educational videos for medical students. However, videos found on YouTube are not peer-reviewed and may be inaccurate, and the preponderance of videos available on the platform makes it difficult for students and educators to find good educational material. We provide a list of URLs of educationally useful videos for students and educators in neurology and offer suggestions for the creation of high-quality educational videos.

## Introduction

An increasing number of medical students use the Internet as their primary source for retrieving and learning new information. Videos are one of the most used virtual education tools and YouTube is the largest online video-sharing platform and the second most visited website after Google Search. Launched in February 2005, YouTube has emerged as an integral part of a medical student’s life [1]. Indeed, unlike books and lectures, its benefits are not limited by time and space. Asynchronous videos have the advantage of presenting up-to-date, digestible information that enables students to revisit topics whenever they want or whenever the need arises during rotations, thereby implicitly incorporating the principle of interleaving as well [2]. Educational videos are particularly useful in the learning and teaching of physical examinations in medicine, especially given that clinical skills cannot be fully learned only by reading textbooks and listening to traditional lectures [3-5]. Videos are also extremely useful in resource-constrained settings, where bedside experience may be limited, as well as in the surgical setting, complementing surgical residents' learning before and after surgery [6-7]. Clinical skills cannot be mastered only by reading books or listening to lectures and require observing clinicians examining patients in real-time. Repeated watching, analysis of gestures, and subsequent practice are important learning strategies facilitated by educational videos on physical examination. Hence, YouTube is a highly beneficial tool for both students and educators in the field of physical examination education [8-9].

However, videos found on YouTube are not peer-reviewed and may be inaccurate and the excessive availability of videos makes it difficult for students and educators to find good educational material [10]. There is no up-to-date study assessing the credibility and usefulness of YouTube videos as a resource for learning neurological examination. In light of this, this study aims to systematically review and assess YouTube videos covering neurological examination to identify educationally useful videos that can be used by students and educators in neurology. We highlight the common drawbacks of the currently available visual material and provide suggestions for the future use and production of educational videos on neurological exams.

## Materials and methods

**Figure 1:**
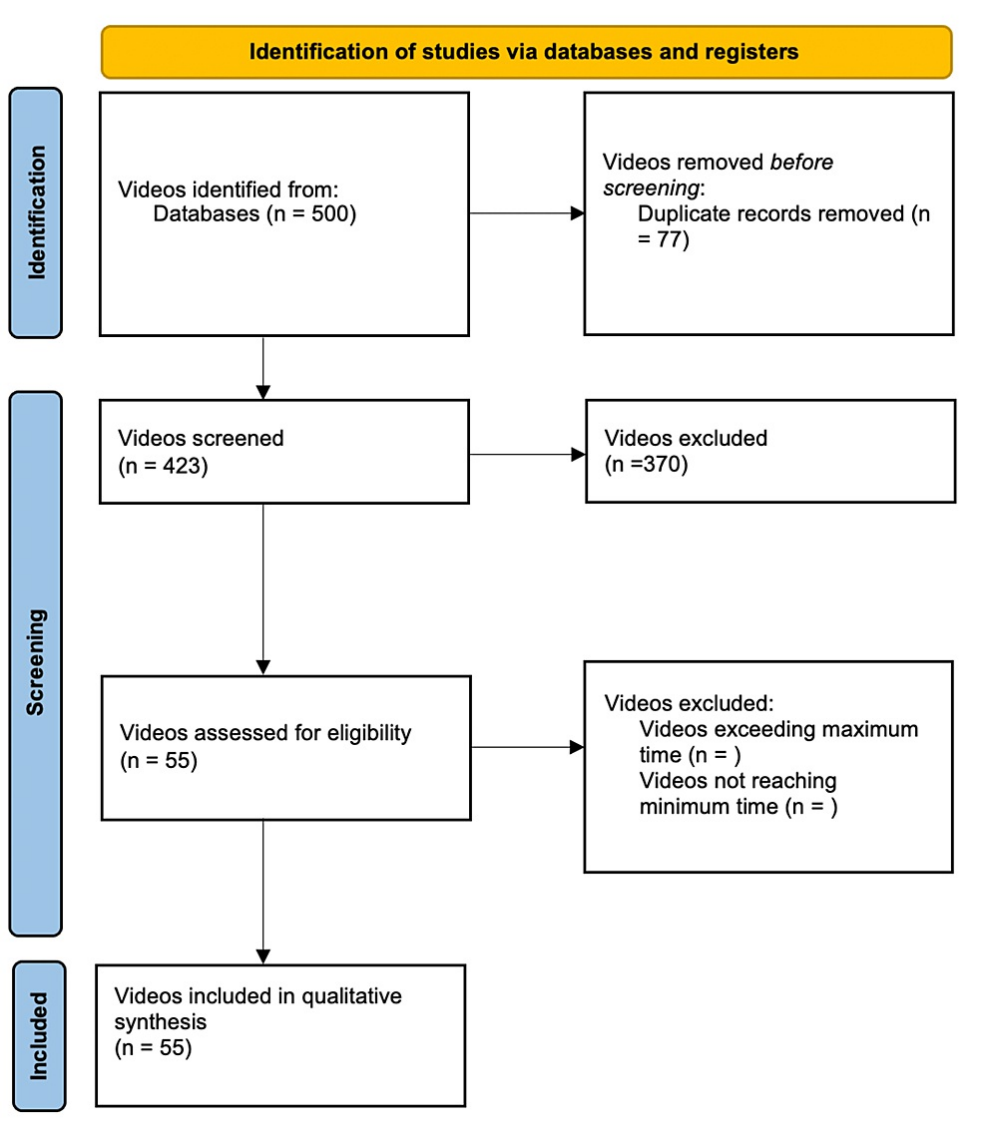
PRISMA flowchart PRISMA: Preferred Reporting Items for Systematic Reviews and Meta-Analyses

From October 2022 to November 2022, we searched Google Videos by using the keywords: “neuro exam”, “neurological examination”, “neurological exam”, “neurological physical examination”, “nervous system examination”, "cranial nerves", "gait", "reflexes". When searching, quotation marks were used with these terms to specify that these terms must be present in the results. Results for each term were screened by title for related videos. We observed that videos not related to search words could already be seen within the first five pages. By pages 7-10, videos not related to the search were found. Videos in the English language posted on YouTube were identified and each URL was recorded. The time frame for video publication on YouTube was not used as an exclusion criterion. The search was conducted by two authors independently using the search keywords (Figure 1). This study complied with the guidelines of the Preferred Reporting Items for Systematic Reviews and Meta-Analyses (PRISMA) as applicable to educational videos [11]. The search results were analyzed and entered into a common pool that was used for further analysis. The inclusion criteria were videos covering clinical examination of the nervous system in adults.

Videos were excluded if they were (1) not in the English language, (2) an advertisement or news, (3) discussing signs or symptoms of diseases affecting the nervous system, (4) about patients with nervous disorders, (5) published on platforms other than YouTube, (6) about drugs used in treating patients with neurological disorders, and (7) in the form of seminars or reviews of the nervous system. Duplicated videos were also excluded and considered as a single item for analysis.

A previously validated scoring system for educational videos [5] was modified according to our subject of investigation and videos were assessed based on 11 items classified into major and minor criteria. The major criteria were as follows: (1) Creator/organization mentioned? (2) Uses simulated or living patient? (3) Images are clear? (4) Sound is clear and the background free of noise? (5) Contents scientifically correct? The minor criteria were as follows: (1) Title is adherent to video content? (2) Designed at the level of medical students? (3) Topic is clearly presented? (4) Drawings, tables, diagrams, animations, or schemas to help retain difficult concepts are used? (5) Is up-to-date information about the author available? (6) Educational objectives are stated?

For each fulfilled major criterion, a score of 2 was allocated. If a major criterion was not fulfilled, no scores were allocated to the video. For each fulfilled minor criterion, a score of 1 was allocated. No half-points were employed. Educationally useful videos were defined as those satisfying all major criteria and at least three minor criteria. Therefore, a score of 13 was used as a threshold to identify educationally useful videos. Scientific accuracy of the content, regarded as a major criterion, was assessed by an experienced neurologist (P.B.). Each video was assessed independently by three medical student investigators. The average of the three independent assessments was taken as the final score for each video. If two videos had the same final score, the standard deviation (SD) was considered.

## Results

A total of 500 videos were identified and assessed for eligibility. After the removal of duplicates and the application of exclusion criteria, a total of 128 videos were included in the final analysis. The average length of the videos was 15 minutes and the total number and average number of views were 10,171,574 and 37,672,496 respectively; 29 videos were about the general neurological exam, while the remaining 99 focused on a specific subtopic. Accordingly, the included videos were classified into the following eight different categories: (1) General neurological exam (29 videos) (Table 1); (2) cranial nerves (23 videos) (Table 2); (3) lectures (28 videos) (Table 3); (4) upper limb (22 videos) (Table 4); (5) lower limb (14 videos) (Table 5); (6) reflexes (six videos); (7) gait (one video); and (8) upper and lower limbs (five videos) (Table 6). Information collected for each video included author details, title, date of upload, and number of views. The number of likes and dislikes was not considered in the analysis since YouTube recently removed the dislike button and some videos do not display the number of likes.

**Table 1:** Characteristics of the included videos on general neurological exam

Video URL	Score	Uploader	Views	Date of upload	Duration, minutes
https://www.youtube.com/watch?v=bMuOqlmf8bo	15.67	Duke Neurology	9,353	06-20-2016	21:02
https://www.youtube.com/watch?v=_iCdCL4pwMs	15.50	UBC Medicine-Educational Media	91,069	02-11-2022	25:31
https://www.youtube.com/watch?v=OgDjOazTt1o	15.33	David Nicholl	20,415	11-02-2016	09:17
https://www.youtube.com/watch?v=r-DtYuOZaiw	15.00	Memorable Psychiatry and Neurology	15,727	06-18-2020	24:09
https://www.youtube.com/watch?v=PEJJv3gJh74	14.67	UCL Clinical Skills	331,336	04-14-2019	20:10
https://www.youtube.com/watch?v=qPzewiv1K74	14.00	Jump Simulation	1,192	01-12-2018	27:49
https://www.youtube.com/watch?v=v0Bz2TF8FCg	14.00	InnOvate	11,299	06-04-2018	16:11
https://www.youtube.com/watch?v=V2MBiS1kc_0	13.67	BilderbackHealth	990,694	08-13-2012	25:43
https://www.youtube.com/watch?v=LKN6pl4vsuE	13.67	World Federation of Neuroscience Nurses	836	11-19-2020	25:03
https://www.youtube.com/watch?v=5UuQV-0o4CE	13.33	Merck Manuals	1,645,225	10-21-2016	05:52
https://www.youtube.com/watch?v=OKz8-OFaxpc	13.33	Prashant	1,338,995	02-11-2014	09:14
https://www.youtube.com/watch?v=ljwEjMShdsw	13.00	Patrick Heyman Zhuravel	229,503	03-08-2018	30:57
https://www.youtube.com/watch?v=Yrt2WRjPdhQ	13.00	ASMR Exams	155,170	01-28-2015	30:53
https://www.youtube.com/watch?v=5IqSw0XdeQY	12.33	Raeburn Forbes	47,171	09-29-2015	04:47
https://www.youtube.com/watch?v=1qkvZaSsGnQ	12.00	University of Dundee Clinical Skills	8,526	08-27-2019	25:21
https://www.youtube.com/watch?v=1WJCbIKO68Q	11.67	getmovingtv	100,030	07-26-2016	10:42
https://www.youtube.com/watch?v=CylQ-Nwwcho	11.33	Robert Dudas	138,099	08-22-2011	14:32
https://www.youtube.com/watch?v=DkrH_6VKPSE	11.00	Nick Smith	31,512	09-02-2016	07:07
https://www.youtube.com/watch?v=dPXsgXwNhDE	10.67	Nursing Assessment and Skills	24,603	01-06-2016	05:57
https://www.youtube.com/watch?v=R_RmlQj-FiU	10.67	Danish Bhatti	4,480,228	04-05-2018	10:53
https://www.youtube.com/watch?v=fgwN1P5PDaA	10.33	Dr Michael Ingram	309,697	01-15-2010	05:22
https://www.youtube.com/watch?v=NcMUTONRGBg	9.67	Medgeeks	18,564	11-07-2018	08:23
https://www.youtube.com/watch?v=Sqb8icF6QhE	9.50	Nursing Assessment and Skills	281,981	10-05-2015	16:37
https://www.youtube.com/watch?v=8f70TOTdmrc	9.33	Juliette Murillo	5,337	11-19-2020	07:46
https://www.youtube.com/watch?v=ZCez3J1wEn0	9.00	Doc J Duenas	11,650	10-16-2020	29:03
https://www.youtube.com/watch?v=ZYUdPUyYV9w	7.67	University of Manitoba Nursing Skills	48,209	11-02-2018	07:20
https://www.youtube.com/watch?v=ml4YGKoCXy4	7.67	Elsevier Australia	49,141	05-16-2015	14:57
https://www.youtube.com/watch?v=7h5o-FJUDRA	7.33	MDforAll	335,148	08-25-2010	05:05
https://www.youtube.com/watch?v=VJjnXeX4OuY	6.00	LoCa's Productions	5,061	11-28-2018	03:29

**Table 2:** Characteristics of the top five educationally useful videos about cranial nerves

Video URL	Score	Uploader	Views	Date of upload	Duration, minutes
https://www.youtube.com/watch?v=CU5Mz18U3jQ	16	UBC Medicine–Educational Media	2,684,208	06-29-2018	18:35
https://www.youtube.com/watch?v=BKOcqlZnCIM	15	Ninja Nerd	1,042,725	04-08-2021	42:49
https://www.youtube.com/watch?v=LkJ4ue00VAI	15	Moran CORE	2,414,517	02-08-2018	16:43
https://www.youtube.com/watch?v=nLsv38ybGJE	15	Dr James Gill	4,363,498	01-26-2020	10:11
https://www.youtube.com/watch?v=arwGZ44C7xM	15	Geeky Medics	519,099	03-06-2012	08:56

**Table 3:** Characteristics of the top five educationally useful videos in the form of lectures

Video URL	Score	Uploader	Views	Date of upload	Duration, minutes
https://www.youtube.com/watch?v=I_JCg8DHO2o	15	AANS - Neurosurgery	17,419	04-23-2012	15:45
https://www.youtube.com/watch?v=dgI65ihSc7o	15	Dr James Gill	253,052	03-07-2021	29:59
https://www.youtube.com/watch?v=gfxvKrurC0M	14	Paramedicine Com	2,431	02-22-2019	16:53
https://www.youtube.com/watch?v=eO8YmD39hkg	14	Know Neuropsychology	620	05-15-2022	59:21
https://www.youtube.com/watch?v=MRJ01G46mj4	13	DrHardik Mistry	156,575	07-17-2019	16:37

**Table 4:** Characteristics of the top five educationally useful videos about the lower limb

Video URL	Score	Uploader	Views	Date of upload	Duration, minutes
https://www.youtube.com/watch?v=HtA_JtD1JQM	15	Chris Lendrumcoach	16,283	04-02-2021	10:50
https://www.youtube.com/watch?v=r85qkj8aRAk	15	UCD Medicine	86,022	01-09-2013	11:10
https://www.youtube.com/watch?v=kZgTq5ZPBiQ	15	Ninja Nerd	198,478	05-03-2021	36:51
https://www.youtube.com/watch?v=quSoCux8k5s	14	Mayo Clinic	278	05-17-2023	17:36
https://www.youtube.com/watch?v=DWKS8rJIwV4	13	Doctor Khalid	582,723	12-17-2014	08:48

**Table 5:** Characteristics of the top five educationally useful videos about the upper limb

Video URL	Score	Uploader	Views	Date of upload	Duration, minutes
https://www.youtube.com/watch?v=0hhcxaeOCYs	16	Geeky Medics	3,054,578	02-17-2015	07:40
https://www.youtube.com/watch?v=JP2rVUeuuK4	15	Ninja Nerd	243,816	04-26-2021	36:27
https://www.youtube.com/watch?v=S7H1pqRlVqc	15	Geeky Medics	390,508	08-06-2011	09:24
https://www.youtube.com/watch?v=z6FBM8u9VZk	15	Dr James Gill	803,758	02-11-2021	07:18
https://www.youtube.com/watch?v=oR7y7995Exk	14	Mayo Clinic	955	05-17-2023	23:58

**Table 6:** Characteristics of the educationally useful videos about the upper and lower limb, gait, and reflexes

Video URL	Score	Uploader	Views	Date of upload	Duration, minutes	Category
https://www.youtube.com/watch?v=jvwAGd1cQh4	16	UBC Medicine–Educational Media	185,461	06-29-2018	08:37	Gait
https://youtu.be/0sqCIzuotWo	15	Stanford Medicine 25	2,879,076	03-17-2014	06:48	Reflexes
https://www.youtube.com/watch?v=oRIkwEUi1Ow	15	Dr James Gill	814,268	06-11-2011	08:41	Reflexes
https://www.youtube.com/watch?v=dMV5UJDbhN0	15	Dr James Gill	162,225	03-18-2021	21:01	Reflexes
https://www.youtube.com/watch?v=dgI65ihSc7o	14	Dr James Gill	253,052	03-07-2021	29:59	Upper and lower limbs

A total of 55 educationally useful videos were identified and listed according to their score. Specifically, 13 were about the general neurological exam, 10 about cranial nerves, 11 about the upper limb, 11 in the form of lectures, five about the lower limb, three about reflexes, one about upper and lower limbs, and one about gait.

Regarding the videos focusing on the general neurological examination (Table 1), six of the educationally useful videos were created by academic institutions; three videos were from online resources of medical books (“Macleod’s Clinical Examination”, “Harrison’s Principles of Internal Medicine”, “Neuroanatomy through clinical cases”) reposted by online users; one by an online medical manual; one video was created by a medical education channel; one video was created by the World Federation of Neuroscience Nurses; and one video was uploaded by an independent medical provider. The top three educationally useful videos were all created by Academic Institutions. The three videos displaying book-related content were published by users other than the video creator or book editor, which raised concerns about regulatory and copyright requirements.

When ordered according to the number of views, only six of the 10 most viewed videos were educationally useful. Moreover, the top three educationally useful videos were not among the top 10 most viewed videos, underscoring the importance of peer review in the evaluation of educational videos. The scientific content and examination routine used in the videos were overall homogeneous. However, we observed that none of the analyzed videos included the evaluation of the autonomic nervous system as part of the physical examination routine. This finding highlights the lack of resources for medical students in terms of learning this aspect of the neurological examination.

## Discussion

This study examined the YouTube videos on neurological examination. Through a systematic search and assessment of videos, we identified 55 educationally useful videos for students and educators of Neurology, 13 of which focused on the general neurological examination. Of note, less than half of the videos screened were determined to be educationally useful, underscoring the importance of a vigorous selection process before their use in medical education. Moreover, the top three educationally useful videos were not among the top 10 most viewed videos. This finding may be explained by the vast amount of video materials available on YouTube and the consequent time-consuming nature of systematically and accurately searching the database.

We found that most of the educationally useful videos were created by academic institutions, highlighting the important role of universities, not only in directing students to the most scientifically accurate educational visual resources but also in producing these contents. Faculty members should be open to new teaching modalities, being cognizant of the changes in the learning behavior of students worldwide [8,12-13]. Therefore, in the creation of useful educational videos, the collaboration between students and teachers may play a key role. Once the decision to incorporate videos in medical teaching has been made, kick-off meetings, including all the figures involved in the learning process, may prove to be useful in deciding which topics would benefit from the use of educational videos and further devise the steps needed to produce and incorporate videos into lectures. Classes need to be redesigned to enable the optimal use of video content and maximization of their educational benefits. Videos can be used to deliver traditional lectures before class (so that more time can be spent on student-centered or practical and interactive activities), and they can also be embedded into lectures or assigned as review/supplementary material [14].

After the planning, the next phase involves the recording of the video content followed by peer review and editing of the visual material produced. Editing is an invaluable part of educational video production and enables the exploration of new avenues to maximize educational benefits and involve students in active learning. Indeed, videos should be designed to manage cognitive load, maintain attention, and facilitate memorization [15]. Mnemonics, on-screen summaries and schemas, images, and illustrations may be employed to facilitate the learning process. Thus, collaboration with technicians, professional video editors, and medical illustrators brings huge benefits to the creative process. The duration of the videos should be based on evidence gathered regarding students’ attention levels [16].

The benefit of active learning afforded by visual materials’ use in medical education is based on the premise of motivation for self-directed learning. However, motivation for self-directed active listening and participation in lectures is a comparable limitation of traditional educational strategies. Moreover, a recent study tried to quantify the level of motivation for self-directedness and found that self-directed learning outperformed traditional instructed learning in an anatomy class [17]. Future studies will need to assess self-directedness in different contexts and with different video usages, further analyzing the variables that may improve the students’ compliance to achieve optimal educational efficacy.

Finally, we have stated that the autonomic nervous system exam was not included in the analyzed educational videos. The examination routine used in the videos was overall pretty homogeneous and we believe this is due to the long-standing unchanged tradition of physical examination in the neurological field. Neurology, more than other branches of internal medicine, still solidly relies on physical examination, rendering it an important tool for the neurologist. Autonomic medicine is still a young subfield, which may explain its lack of inclusion in the analyzed videos. Given the scarce attention paid to medical schools on the topic of autonomic neurology, we believe that video creation can represent a tool and a future area of intervention for leaders in the neurology educational field to shape and direct attention to this topic in the medical school curricula [18-21].

This study has a few limitations. YouTube is a dynamic and extremely popular platform and scores of videos are uploaded on it unceasingly, which shows the necessity of new studies in the future to update the snapshot provided herein. Moreover, we considered only English-language videos published on YouTube. More educationally useful videos published on other websites or created in other languages may be analyzed in future research. The creation of English subtitles may solve the language barrier problem, hereby enabling future studies to focus on non-English language videos as well.

## Conclusions

YouTube is an exceedingly common source of educational videos for medical students. However, videos found on YouTube are not peer-reviewed, may be inaccurate and the abundance of videos available makes it difficult for students and educators to find good educational material. We provided a list of URLs of educationally useful videos for students and educators in Neurology. Our results show that the most viewed videos are not the most educationally useful and underscore the importance of academic institutions in the creation of valuable educational video materials.
